# The Feasibility of Achieving Low-Sodium Intake in Diets That Are Also Nutritious, Low-Cost, and Have Familiar Meal Components

**DOI:** 10.1371/journal.pone.0058539

**Published:** 2013-03-07

**Authors:** Nick Wilson, Nhung Nghiem, Rachel H. Foster

**Affiliations:** Department of Public Health, University of Otago, Wellington, New Zealand; The University of Manchester, United Kingdom

## Abstract

**Objective:**

Given the importance of high sodium diets as a risk factor for disease burden (ranked 11^th^ in importance in the Global Burden of Disease Study 2010), we aimed to determine the feasibility of low-sodium diets that were also low-cost, nutritious and (for some scenarios) included familiar meals.

**Methods:**

The mathematical technique of “linear programming” was used to model eight optimized daily diets (some with uncertainty), including some diets that contained “familiar meals” for New Zealanders or were Mediterranean-, Asian- and Pacific-style diets. Data inputs included nutrients in foods, food prices and food wastage.

**Findings:**

Using nutrient recommendations for men and a cost constraint of <NZ$9/d (US$6.84), the sodium intake levels in the eight optimized daily diets were all well below the 2300 mg/d (5.8 g salt/d) recommended maximum. The only diet to not consistently fall below the recommended “target” upper limit of 1600 mg/d included an evening meal with sausages (median  = 1640 mg/d, 95% simulation interval: 1551–1735 mg/d). Many additional nutritional aspects of these optimized low-sodium diets suggest that they would reduce cardiovascular disease risk in other ways (e.g., improved polyunsaturated to saturated fat ratio) and also reduce risk of cancer and other chronic diseases (e.g., via higher intakes of vegetables, fruits and dietary fiber). Even healthier diets (e.g., with higher intakes of fruit) occurred when the cost constraint was relaxed to $NZ15/d (US$11.40). Similar results were obtained when the modeling considered diets for women.

**Conclusions:**

These results provide some reassurance for the feasibility of substantially reducing population sodium intake given currently available low-cost foods and while maintaining some level of familiar meals. Policy makers could consider ways to promote such optimized diets and foods, including regulations on maximum salt levels in processed foods, and taxes on alternative foods that are high in salt, sugar and saturated fat.

## Introduction

A high intake of salt in the diet is associated with a significantly increased risk of stroke and total cardiovascular disease according to a meta-analysis of 13 observational studies [1]. There is also evidence from a Cochrane systematic review that reductions in sodium intake reduce systolic blood pressure (BP) in people defined as “normotensive” by about 1.3 to 4 mm Hg and even more so in people with hypertension (6 to 10 mm Hg reduction) [2]. In turn, there is a direct relationship between BP and heart disease, stroke and end-stage renal disease according to a meta-analysis of 61 studies [3].

Yet the direct evidence for dietary salt reduction leading to a reduction in disease outcomes is less conclusive. A recent Cochrane systematic review reported that salt reduction had relatively small benefits on cardiovascular events or mortality, and that uncertainty remained [4]. However, this review has been criticized for its approach to study inclusion and other methodological issues [5], [6], [7]. Its low power has also been noted; the analysis only had 10% power to detect a 10% reduction in relative risk [6].

When looking at the totality of the evidence for the potential benefits to health from the reduction of dietary salt intake, there is also evidence from many animal studies, ecological studies and observational studies [8], [7]. Particularly notable improvements in cardiovascular health associated with dietary salt reduction were achieved in Finland after institution of systematic approaches to reduce salt intake across the population, such as mass media-campaigns, co-operation with the food industry, and implementing salt labeling legislation. Nevertheless, other cardio-protective changes may also have played important roles in these trends e.g., increased potassium intake, increased fruit and vegetable intake, and reduced smoking [7].

In the most recent review we identified was by the World Health Organization (WHO), and the findings were that:

“Higher sodium intake was associated with higher risk of incident stroke, fatal stroke and fatal coronary heart disease. There was no association between sodium intake and all-cause mortality, incident cardiovascular disease and non-fatal coronary heart disease. However, the strong positive relationship between blood pressure and these outcomes provides indirect evidence that reducing sodium intake can improve these outcomes through a beneficial effect on blood pressure.” [9]

As others have commented on, it is clear that there are considerable complexities in considering the evidence around dietary salt for policy development [10]. Nevertheless, given the totality of the currently available evidence it seems highly justifiable for governments and health authorities to act further on lowering population sodium intakes. The need for this may even grow more urgent if publicly-funded health systems come under additional fiscal pressure for treating cardiovascular disease in older populations. For these reasons the WHO in 2012 recommended a “reduction to <2 g/day sodium (5 g/day salt) in adults (*strong recommendation*)” [9].

Certainly at the international level there are calls for “salt reduction” to be considered a priority, with it included in the top five priority actions for advancing non-communicable disease (NCD) control internationally [11]. “High sodium” is also one of the top two dietary risk factors for disease burden identified in the Global Burden of Disease Study 2010 [12]. It was ranked 11^th^ globally out of all risk factors, and was ahead of all other dietary risk factors except for diets “low in fruits”.

Yet to facilitate the introduction of salt reduction interventions, it is still useful to address any residual concerns that lower sodium diets might carry health risks and are not particularly feasible. Fortunately there is minimal evidence for risks associated with reducing salt intake, as noted in a major report by the Institute of Medicine (USA) that made recommendations for regulating down the salt levels in foods [13]. Similarly, the WHO Review stated that “Reducing sodium intake had no significant adverse effect on blood lipids, catecholamine levels or renal function” [9]. But in terms of feasibility, it has been suggested for the US population that meeting salt reduction goals would require a potentially unfeasible deviation from current eating patterns or a profound modification of the US food supply [14]. Furthermore, optimized diets (that are low sodium, nutritious and low cost) may potentially have relatively little variety and deviate “substantially from social norms” as suggested in a French study [15].

Given this background, this study aimed to further determine the feasibility of diets that meet not only nutritional recommendations for sodium (≤2300 mg/day [d] or ≤5.8 g salt/d) but were also low-cost, nutritious and (for some scenarios) included familiar meals for the New Zealand population.

## Methods

To avoid existing problems with poor nutrition in the New Zealand diet, this analysis took a “bottom-up” approach by obtaining data on a wide range of individual food items and optimizing towards a daily diet meeting low sodium and other nutritional requirements from there. Nevertheless, other scenarios were generated that contained “more familiar meals” (to New Zealanders) along with Mediterranean-, Asian- and Pacific-style diets. The mathematical technique of linear programming was applied to define the optimal solutions for eight different daily diets.

### Nutrient Constraints

Diets were modeled to meet average requirements for key macronutrients and micronutrients included in the New Zealand Adult Nutrition Survey (NZANS) [16]. The “estimated average requirements” (EARs) of nutrients, or minimum levels for adequate intake, for Australia and New Zealand from the (Australian) National Health and Medical Research Centre were used in most cases [17].

A conservative approach was taken by modeling nutrient requirements for only men in the baseline models (since it is more difficult to achieve low sodium intakes for men who have generally higher food intakes due to dietary energy demands). However, the baseline models are still relevant for women because nutrient requirements for women are the same or less than those for men with the exception of iron. To address this, a constraint for iron such that the EAR value for women (8 mg/d) was used rather than the value for men (6 mg/d). Nevertheless, additional modeling using the nutrient requirements for women was also performed.

As per recommendations, the upper limit for sodium was set at 2300 mg/day [d] [17], which is equivalent to 5.8 g of salt/d. The minimum level for sodium intake was set at 460 mg/d. Upper limits were also set for saturated fatty acids (10% of daily energy, approximately 30 g for men), and vitamin A (3000 µg/d). Minimal levels were set for polyunsaturated fat (13 g/d for men), protein (52 g/d for men) and fiber (30 g/d for men), and the selected minerals and vitamins. For simplicity, this analysis included only one of the B vitamins, thiamine (B1), for which intakes are most inadequate in the New Zealand setting [16]. Details are provided in Table S1 in File S1.

### Dietary Scenarios

Two daily food cost constraints of <NZ$9/d (US$6.84) and <NZ$15/d (US$11.40) were applied to all dietary scenarios. The former reflects data from an annual survey (the University of Otago “Food Cost Survey”) where for 2011 the calculated costs for a “basic diet” were NZ$9.29/d for men and NZ$8.71/d for women [18].

In the initial stages, our low-sodium optimization analyses had cost constraints but no upper limits for dietary energy. However, the results often involved large excesses in energy intake as the optimization process strove to obtain adequate micronutrients (particularly calcium and vitamin A) from very low cost foods. As a result we modified our scenarios (to those shown in Table S2 in File S1) and focused on low-sodium optimization for a set level of daily energy of 11,450 kJ (2734 kcal) for men. This represents the estimated energy requirement averaged for four male adult age-groups at the mid-range level of physical activity of 1.7 MJ/d [17].

In all scenarios, the daily maximum limit for any single high-carbohydrate food (e.g., flour, pasta, rice, oats, couscous) was 120 g, other than a requirement for > = 200 g of rice for the Asian diet scenario. No more than 200 g of any particular vegetable or fruit (excluding starchy root crops: potatoes, taro and kumara) was permitted. Other limits are shown in Table S2 in File S1. Lower limits for foods were not set given that this greatly complicated the optimization programming and small amounts of specific foods are routinely used in modern daily diets (e.g., in salads, stews, stir-fried meals etc).

The first scenario (“BASIC1”) focused on achieving the lowest daily sodium intake (albeit to a minimum of 460 mg/day) while meeting recommended nutrient concentrations (Table S2 in File S1). The next scenario (“BASIC2”) was identical to BASIC1 but made use of more familiar (but low cost) basic meal components: porridge with milk, and roti (naan/flat bread) utilizing low-cost flour.

We then considered two scenarios of dietary patterns with aspects that are likely to be health-promoting: a Mediterranean-style diet (“MED”) including fish/seafood, olive oil and a high intake of fruit and vegetables [19] and an Asian-style diet (“ASIAN”) including rice, oil for stir-fry cooking and a relatively high intake of fruit and vegetables. However, we excluded the typically high-salt Asian sauces. These types of diets were selected because there are now many systematic reviews favoring the impact of vegetable and fruit consumption on health (preventing various cancers [20], [21], [22]; type 2 diabetes [23]; stroke [24]; and coronary heart disease [25]). Furthermore, systematic reviews also indicate health benefits of the Mediterranean diet for preventing major chronic diseases [24], [26], [27], [28]. Also of note is that Asian-style diets are of increasing relevance to New Zealand with the growing Asian population in the country, including the growing popularity of restaurants selling Asian-style food.

For scenarios which included “more familiar meals” (potentially more acceptable to New Zealanders), evening meals were selected that were likely to be relatively low-cost. The selected meals were: main meal – mince involving mince on toast (Scenario “NZ-Meat1”); main meal – sausages that also included potatoes and an ice-cream with canned fruit dessert (“NZ-Meat2”); main meal – tuna pasta (“NZ-Fish”), and; main meal – Pacific style that included tuna, taro and coconut cream (“NZ-Pacific”) (see Table S2 in File S1). The Pacific style meal was selected because Pacific Peoples are a growing part of the New Zealand population. All of these scenarios also included a range of fruit and vegetables, a low-cost breakfast (porridge with milk) and a low-cost lunch (cheese sandwich, peanut butter sandwich and an apple). For these meals readily available recipes were used e.g., for the Pacific style evening meal, a recipe was used from the Food and Agriculture Organization of the United Nations website (http://www.fao.org/WAIRdocs/x5425e/x5425e01.htm) and for the mince meal a recipe on the “NZ Beef and Lamb” website was used.

### Selection of Food Items

To simplify the number of food item options to be included in the modeling, only foods used in compiling the country’s Food Price Index (FPI) [29] were initially used (n = 44 commonly purchased food items). But to expand the range of low-cost foods we also included foods from: (i) previous work that identified low-cost sources of protein in New Zealand [30]; (ii) unprocessed foods (e.g., lentils and peanuts) commonly found in the “bulk bins” at the supermarket and low-cost canned foods (convenience sample in the capital city, Wellington); (iii) lists of selected foods from a previous nutrition optimization study in France [15]; and (iv) foods not covered above but which were needed to fit with recipes for the lunch and evening meals in the scenarios orientated to “more familiar meals” for New Zealanders (e.g., the starchy vegetable “taro” for the Pacific style meal).

This process resulted in a total of 76 food items (see Table S3 in File S1). To maximize potential health benefits we ensured that in the scenarios with “fixed meal components” we included relatively healthier variants e.g., unsalted nuts, wholemeal flour, wholemeal bread, low-salt margarine, low-fat ice cream, and “lite” coconut milk. Nevertheless, white rice was included rather than wholegrain rice given that the former is very much more popular in New Zealand and involves much shorter cooking times.

### Food Price and Nutrient Inputs

For most of the food items, Food Price Index (FPI) price data were used (monthly data averaged over multiple stores nationally for the 12 months of 2011) [29]. However, where food items were not covered in the FPI, online supermarket data were used (Countdown, January 2012), or the lowest in-store (e.g., bulk bin) prices from New World or Countdown supermarkets (both in Karori, Wellington). A conservative approach was taken by ignoring prices on “specials” and set the maximum size for food product pricing at 1.5 kg (i.e., generally avoiding savings from bulk purchase). In the Asian scenario only, a supermarket price for bulk rice of $17.99 for 10 kg was used.

Nutrient values for the foods were obtained from the 2012 “New Zealand food composition database” (http://www.foodcomposition.co.nz/foodfiles). Estimated nutrient intakes were adjusted to account for food wastage. As detailed food wastage data are not available for New Zealand, the values used were from a large study on food wastage (the WRAP study) from the United Kingdom (UK) [31].

### Typical New Zealand Diet

To allow for comparisons, we also modeled our best estimate of the typical New Zealand diet (for men). This utilized national survey data (NZANS) [16] that provided data on the proportional contribution of dietary energy intake for different food categories (excluding alcohol). To each of these categories we assigned in varying proportions relevant food items for which we had assembled price and nutrient data (n = 76 food items as described above; spreadsheets for workings available on request). This method gave a total of 9996 kJ of dietary energy and so we then scaled the results to the 11,450 kJ intake used in the other analyses. We then calculated the sodium intake and daily cost associated with these foods.

### Mathematical Modeling

The “simplex algorithm” was used to solve the linear programming problem (see Briend et al [32] for a detailed description of linear programming in the nutritional context). Most of the scenarios were modeled in Microsoft Excel 2010 (Excel Solver, Simplex method). However, R programming language (version 2.15.0, lpSolve package) was used where there was a high level of complexity with the food combination options (e.g., selections to achieve a certain level of fruit and vegetables). In the instances where it was possible to verify, both approaches produced near identical results.

### Uncertainty and Heterogeneity

Uncertainty in food prices was incorporated using the variation in the monthly prices from the FPI data, and fitting this to gamma distributions. For non-FPI foods the same patterns observed for the FPI foods in the same food category were applied (e.g., from the median values of the “fresh fruit and vegetable” grouping).

The variability of nutrient content of foods (e.g., by variety or brand and level of freshness) was addressed by applying to all nutrient values a normal distribution with a standard deviation (SD) equal to ±5% of the mean value.

There is substantial uncertainty around food wastage including waste arising from how food is stored, eating habits, and size of food products (e.g., purchase of larger sized items might lead to relatively more waste [33]). To address such uncertainty for the total food waste proportion, the SD calculated from the UK food waste study, the “WRAP study” [31], were used to specify a beta distribution. For the food items where there was no clear match between the WRAP study and our database, the median SD of all the matched food items was used.

To account for population heterogeneity in nutrition, we used the distributional data identified in average nutrient requirements for different types of men (of differing sizes and activity levels) for Australia and New Zealand [17]. However, for the target energy intake we derived distributional values from the published survey results (based on the 95%CIs in the NZANS [16]; we assumed a normal distribution with SD  = 184.4).

We then coded the models and ran 2000 iterations for representative scenarios in R programming language (version 2.15.0). As we modeled probabilistic distributions for both uncertainty and heterogeneity, we use the term simulation intervals in the combined final output.

## Results

Using a food cost constraint of <NZ$9/d (Table 1), the sodium intake level for the optimal solution ranged from the constrained lower limit of 460 mg/d (Scenarios BASIC1, BASIC2, “MED” and “ASIAN”) to between 899 and 1641 mg/d (for the four scenarios with more familiar meal components). That is, all were well below the 2300 mg/d recommended upper limit for sodium and only one scenario (Scenario NZ-Meat2) exceeded the recommended “target” upper limit of 1600 mg/d [17]. Overall, vegetables, fruit and cereals and grains were particularly selected in the scenarios with relatively small amounts of diary products and meat (except where this was required in the “familiar meal” components).

**Table 1:** Foods selected by the optimization process for men for low-sodium and for the various daily dietary scenarios with a daily cost constraint of <NZ$9/d.

	Dietary Scenario							
Food (g/d)	BASIC1	BASIC2	MED	ASIAN	NZ-Meat1	NZ-Meat2	NZ-Fish	NZ-Pacific
**Vegetables** a	Total 193 g	Total 233 g	Total 549 g	Total 500 g	Total 253 g	Total 200 g	Total 352 g	Total 296 g
	*Mushrooms, carrots, tomatoes*	*Mushrooms, carrots*	*Chinese cabbage, peas* *(F), carrots, mushrooms,* *onions*	*Chinese cabbage, cabbage, onions,* *broccoli, carrots*	*Peas (F), onions, carrots*	*Carrots*	*Tomatoes (C), fresh tomatoes, carrots*	*Peas (F), cabbage, carrots, onions*
**Starchy vegetables**	Potatoes 186 g	Potatoes 180 g	0	Potatoes 34 g	0	Potatoes 426 g	0	Taro 104 g
**Fruit** a	0	Total 5 g	Total 200 g	Total 82 g	Total 551 g	Total 277 g	Total 595 g	Total 330 g
		*Oranges*	*Oranges*	*Apricots (C)*	*Apricots (C), oranges, apples, peaches (C)*	*Apricots (C), apples*	*Apricots (C), peaches* *(C), apples, oranges*	*Apricots (C), apples*
**Cereals and grains** b	Total 565 g	Total 623 g	Total 200 g	Total 579 g	Total 382 g	Total 327 g	Total 414 g	Total 367 g
	*Pasta, white rice, semolina, couscous,* *oats, wheat germ*	*Pasta, white rice,* *white flour, couscous,* *oats, semolina*	*White flour, semolina*	*White rice, pasta, couscous, semolina,* *oats, white flour*	*Oats, white flour, bread, couscous, wheat germ*	*Couscous, white flour, bread, oats*	*Oats, white flour, pasta, bread*	*Oats, white flour, bread, semolina, wheat germ*
**Pulses, seeds** **and nuts**	Sunflower seeds 21 g	Sunflower seeds9 g	Peanuts 163 g, Sunflower seeds 23 g	Sunflower seeds 10 g	Sunflower seeds 70 g	Sunflower seeds 70 g	Sunflower seeds 70 g	Sunflower seeds 70 g
**Meat/fish**	0	Pork chops 6 g	Sardines 24 g	Pork mince 93 g	Beef mince 125 g	Sausages 96 g	Tuna 124 g	Tuna 77 g
**Dairy**	Milk (H) 244 g, milk powder 27 g	Milk powder 42 g	Milk powder 27 g	Milk powder 42 g	Milk powder 25 g	Milk powder 25 g	Milk powder 25 g	Milk powder 25 g
		Cheese 14 g		Cheese 3 g	Cheese 12 g	Cheese 12 g	Cheese 12 g	Cheese 12 g
						Ice-cream 66 g		
**Added fat/spreads**	“Lite” coconut cream 56 g	Olive oil 17 g	Olive oil 60 g	Vegetable oil 14 g	Peanut butter 13 g	Vegetable oil 25 g	Peanut butter 13 g	“Lite” coconut cream 222 g
	Butter 30 g	Margarine 16 g	“Lite” coconut cream5 g	Olive oil 10 g	Margarine 10 g	Peanut butter 13 g	Margarine 10 g	Peanut butter 13 g
	Olive oil 13 g	Peanut butter 4 g			Vegetable oil 7 g	Margarine 10 g		Vegetable oil/margarine 23 g
**Other**	0	0	Eggs 68 g	Eggs 37 g	Sugar 7 g	Sugar 7 g	Sugar 7 g	Sugar 7 g
**Total food**	**1337 g/d**	**1150 g/d**	**1318 g/d**	**1404 g/d**	**1455 g/d**	**1553 g/d**	**1622 g/d**	**1546 g/d**
**No. items**	**16**	**17**	**15**	**20**	**20**	**17**	**19**	**21**
**Sodium**	**460 mg/d**	**460 mg/d**	**460 mg/d**	**460 mg/d**	**905 mg/d**	**1641 mg/d**	**1101 mg/d**	**899 mg/d**

a Presented in descending order of quantity.

b Wholemeal or wholegrain unless otherwise stated.

C = canned; F = frozen; H = homogenized.

These modeled dietary scenarios would appear to be closer to recommended nutrient intakes than the typical New Zealand diet for men in terms of being: lower in saturated fats (all), higher in polyunsaturated fats (in 5/8 scenarios); lower in total sugars (6/8); and higher in: dietary fiber (all), potassium (all), iron (7/8), and vitamin E (5/8) (Table 2). The modeled diets also had more favorable ratios of potassium to sodium (all), and polyunsaturated to saturated fatty acids (7/8 scenarios). The Mediterranean diet appeared to be the one providing the closest results to the recommended nutrient intakes (i.e., albeit only slightly better results than for the ASIAN diet). Of note however, was that the range of foods in these eight dietary scenarios was limited to between 15 and 21 items.

**Table 2:** Sodium and other nutrient intakes for the optimal solution for men and for the various daily dietary scenarios with a cost constraint of <NZ$9/d (where sodium was the objective function value in each scenario).

	Dietary scenario								
Nutrients (constraints)	BASIC1	BASIC2	MED	ASIAN	NZ-Meat1	NZ-Meat2	NZ-Fish	NZ-Pacific	Typical NZ diet^a^
Cost (<NZ$9)	8.99	8.99	8.99	8.99	8.99	8.66	8.99	8.99	17.29^b^
Energy ( = 11,450 kJ)	11,450	11,450	11,450	11,450	11,450	11,450	11,450	11,450	10,380
Saturated fatty acids (≤30 g)	30	12	30	9	24	20	12	30	36.5
Polyunsaturated fatty acids (≥13 g)	13	13	40	13	36	39	31	37	13.1
Protein (≥52 g)	93	99	108	109	118	92	128	114	102
Total sugars (g)	26	23	46	60	127	96	146	103	120
Dietary fiber (≥30 g)	30	30	42	30	65	37	62	66	22.1
**Selected minerals**									
Sodium (≥460 and ≤2300 mg)	460	460	460	460	905	1641	1101	899	*2970* c
Potassium (≥3800 mg)	3800	3800	3800	3800	4394	5024	4382	4399	3449
Calcium (≥840 mg)	840	840	840	840	840	840	840	840	919
Iron (≥8 mg)	14	15	15	12	32	18	29	29	13.2
Zinc (≥12 mg)	12	12	13	12	26	13	18	25	12.9
Selenium (≥60 µg)	60	60	60	60	60	69	119	86	67
**Selected vitamins**									
Vitamin A (≥625 & ≤3,000 µg RE)	625	625	1177	754	625	2290	625	625	846
Thiamine (≥1 mg)	2	2	3	2	5	3	4	4	1.6
Vitamin C (≥30 mg)	30	30	152	138	110	80	101	80	99
Vitamin D (mcg)	3	3	4	1	2	2	7	5	-
Vitamin E (≥10 mg)	12	10	32	10	40	37	38	42	11.5
**Calculated ratios**									
Polyunsaturated/saturated fats ratio^d^	0.4	1.1	1.3	1.4	1.5	2.0	2.6	1.2	0.4
Potassium/sodium ratio^d^	8.3	8.3	8.3	8.3	4.9	3.1	4.0	4.9	1.2

a Self-reported intake for men from the New Zealand Adult Nutrition Survey (NZANS) 2008/2009 [16] unless otherwise stated.

b Estimate based on our modeling of 76 possible food items using either Food Price Index cost data or the lowest cost item available.

c Conservative estimate based on our calculations for the typical New Zealand diet, excluding discretionary salt and preferentially selecting certain lower salt options (see *Methods*).

d Ratios of mean (and median and SI), not the mean ratio.

RE – retinol equivalents.

When the upper cost limit was relaxed to NZ$15/d, the sodium level in the optimized diets was similar or slightly decreased (i.e., lower in scenarios: NZ-Meat1, NZ-Fish, NZ-Pacific) (see in Table S4 in File S1 for the listed foods and Table S5 for the nutrient concentrations). One of the most notable differences between the optimal solutions for the $9/d and $15/d constraints was the increased fruit consumption in the latter (in 7/8 scenarios) and to a lesser extent vegetable consumption (3/8 scenarios). As for the lower cost constraint, all these diets would probably be healthier than the typical New Zealand diet for men (e.g., often with even more favorable potassium to sodium ratios).

**Figure 1:**
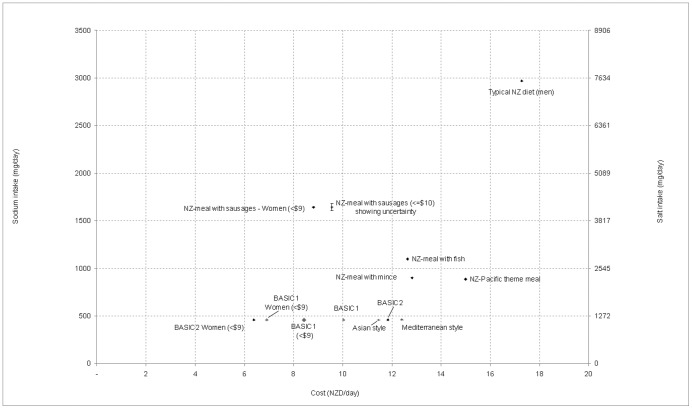
Sodium intake and cost of the various daily dietary scenarios as a result of the low-sodium optimization process (all optimized for <$15/d for men unless otherwise indicated) and compared with the typical diet for a New Zealand man*. *Note: The sodium intake for typical New Zealand diet was based on a conservative estimate excluding discretionary salt and preferentially selecting certain lower salt options (see *Methods*).

As shown in Figure 1, all the scenarios for the $15/d cost constraint had sodium intakes well below our conservative modeled estimate of 2970 mg/d for a typical New Zealand diet for a man, as well as being lower in cost. Indeed, the gap is likely to be even greater as our modeling assumptions for this “typical diet” did not include the addition of discretionary salt and may partly reflect our approach of selecting “healthy” food variants (e.g., low-salt margarine) for inclusion in our dataset. Our estimate is not dissimilar to a recent estimate for New Zealand men of 2901 mg/d, which also excluded consideration of discretionary salt [34]. But this estimate is lower than the 4013 mg/d for New Zealand men based on spot urine data from a national nutrition survey [35]. The cost of this typical diet (at $17.29/d) was also substantially more expensive than the low-sodium diets (Table 1, and also Table S4 in File S1).

The uncertainty and heterogeneity analysis relating to the foods selected by the optimization process are shown for the lowest sodium diet (Scenario BASIC1) and the highest (NZ-Meat2) for the $9/d constraint in Table 3. The most commonly selected foods were similar to those found in deterministic analysis (Table 1) but there were additional numbers of foods that were included e.g., 20 and 27 foods reflected in the mean values respectively. But smaller numbers of foods were included in the median values at 11 and 18 respectively and in some cases the food was so rarely selected that it did not even appear within the 95% simulation interval (e.g., cabbage in Scenario NZ-Meat2). By definition, there was no uncertainty around values that we forced into the model to create certain meals (e.g., sausages, potatoes, bread, apple, ice cream, cheese and peanut butter for NZ-Meat2 as per Table S2 in File S1). In contrast, the BASIC1 scenario had no forced food items though the upper SI limits could not exceed upper limits set by the model constraints (e.g., 200 g for any particular fruit or vegetable).

**Table 3:** Uncertainty and heterogeneity analysis of foods selected by the optimization process for men for one of the lowest sodium intake daily diet (BASIC1) and the highest sodium intake diet (NZ-Meat2) with a cost constraint of <$9/d.

	A low sodium intake scenario (BASIC1) selected foods (g/day) (2000 iterations)				The highest sodium intake scenario (NZ-Meat2) selected foods (g/day) (403 iterations)^a^			
Food items	Mean	Median	Lower 95%SI bound	Upper 95%SI bound	Mean	Median	Lower 95%SI bound	Upper 95%SI bound
**Vegetables**								
Tomatoes (fresh)	87	0	0	200	79	100	0	100
Carrots	34	35	6	58	200	200	200	200
Cabbage	7	0	0	200	2	0	0	0
Onions	0	0	0	0	14	0	0	100
Oranges	0	0	0	0	11	0	0	184
Bananas	0	0	0	0	5	0	0	91
Peas (frozen)	0	0	0	0	5	0	0	100
**Starchy vegetables**								
Potatoes	84	0	0	354	426	426	426	426
**Fruit**								
Apricots (canned)	194	200	73	200	133	147	0	200
Peaches (canned)	20	0	0	200	16	0	0	147
Apples	0	0	0	0	130	130	130	130
Kiwifruit (green)	0	0	0	0	19	0	0	200
**Cereals and grains**								
Couscous	119	120	108	120	119	120	105	120
Flour (wholemeal)	80	115	0	120	21	0	0	120
Wheat germ	1	0	0	11	8	2	0	29
Bread (wholemeal)	0	0	0	0	56	56	56	56
Oats (whole grain)	0	0	0	0	45	39	39	120
Flour (white)	0	0	0	0	35	0	0	120
**Pulses, seeds and nuts**								
Sunflower seeds	70	70	70	70	63	70	33	70
Peanuts	39	0	0	200	0	0	0	0
**Meat and fish**								
Sardines (canned)	1	0	0	19	0	0	0	0
Sausages	0	0	0	0	96	96	96	96
**Dairy**								
Yogurt	171	250	0	250	0	0	0	0
Milk powder (skim)	45	47	9	82	25	25	25	25
Ice cream	0	0	0	0	66	66	66	66
Cheese	0	0	0	0	12	12	12	12
**Added fat, spreads**								
Olive oil	53	60	13	60	0	0	0	0
Peanut butter	50	15	0	200	13	13	13	13
Butter	13	12	0	34	0	0	0	0
Vegetable oil	1	0	0	2	41	46	0	90
Margarine	0	0	0	0	10	10	10	10
**Other**								
Sugar	28	3	0	60	7	7	7	7
Apple juice	14	0	0	77	0	0	0	0
**Key nutrient: Sodium** b	**460**	**460**	**460**	**460**	**1,644**	**1,640**	**1,551**	**1,735**

SI = simulation intervals from probabilistic distributions for both uncertainty and heterogeneity.

a For this scenario the daily food cost limit had to be raised (up to $10/d) to increase the number of feasible solutions. Even so, the number of feasible solutions was constrained given that we decided to maintain all the upper limits for foods used in the deterministic modeling and the full range of uncertainty and heterogeneity possible (i.e., for nutrient concentrations, nutrient requirements, food prices and food wastage levels).

b See Table S6 in File S1 for the detail on the other nutrients.

For all 2000 iterations it was possible to achieve the lowest sodium level permitted (460 mg) in Scenario BASIC1 (Table 3 and Table S6 in File S1). But for Scenario NZ-Meat2, which included the meal with sausages, the median was 1640 mg/d and the 95% simulation interval was 1551 to 1735 mg/d. Thus, while still always being below the 2300 mg/d upper limit, the sodium level did usually exceeded the “target” upper limit of 1600 mg/d. Uncertainty analysis for other nutrients in presented in Table S6 in File S1.

The results using the nutrient requirements for women are shown in Table S7 for foods and Table S8 for nutrients (File S1). These results indicate that fairly similar foods were selected (compared to those selected for men for the <$9 constraint, as per Table 1), though the food amounts in the various categories were generally less. The reduced energy requirements for the same cost constraint (of <$9) meant that some additional higher cost foods were also selected (i.e., lettuce, kiwifruit, and yogurt). The sodium level results were also very similar to those for men i.e., all considerably below the recommended upper level of 2300 mg and in the range 460 mg to 1642 mg (the latter being for the NZ-Meat2 scenario with the sausage meal).

## Discussion

This study was able to identify a range of low cost dietary patterns (including some with “familiar meal” components in the New Zealand context), that meet low sodium recommendations for adults. Indeed, all of these diets were well under the recommended upper limit for sodium of 2300 mg/d [17]. Nevertheless, one diet did not consistently fall below the <1600 mg/d level (the recommended target upper limit [17]). This was an evening meal that included sausages (Scenario NZ-Meat2) at 1641 mg/d (Table 1). The higher level of sodium with this diet is not surprising given that processed meats are a major source of sodium in the New Zealand diet [36], [34].

As well as the low sodium levels, these diets would also have other cardiovascular disease prevention aspects (compared to the typical New Zealand diet), such as the higher ratio of polyunsaturated to saturated fat intake [37], and possibly also the higher potassium intake (independent of sodium) [38]. Some scenarios were high vegetable diets at over 400 g/d of non-starchy vegetables (e.g., MED, ASIAN), and with the higher cost constraint ($15/d) half of the diets included more than 300 g/d of fruit. Such diets would probably be superior to the current New Zealand diet in terms of preventing various chronic diseases (see the systematic reviews on high fruit/vegetable diets and the Mediterranean diet referred to in the *Methods Section*).

While a US modeling study found that a sodium goal of 2300 mg/d sodium goal was consistent with nutrient adequate diets, it found that achieving lower levels (<1500 mg/d) as recommended for some population groups in the US, was less feasible [14]. Other work on mathematically optimized French diets has shown that diets can be low sodium (<2365 mg/d), meet other nutritional requirements, be low-cost and be culturally appropriate [15]. As with our work, some of these selected French diets even contained foods that are usually relatively significant sources of dietary salt i.e., bread, butter, margarine, canned fish and processed meat products. Importantly, our work demonstrates that some more familiar meals that might normally be seen as less nutritionally desirable (but which are nevertheless popular) can still be incorporated into a low-sodium, nutritionally complete diet when combined with other various more optimal foods.

Our results can also be seen as conservative in terms of what could be achieved in reducing population sodium intake because the modeling is based on current available products, whereas further reductions could be achieved by reformulation of higher salt foods such as bread, processed meats, sauces and various other processed foods.

### Study Strengths and Limitations

A strength of this study is the bottom-up approach that investigated a range of possible dietary scenarios. Dietary optimization analyses can begin with typical dietary patterns and explore incremental shifts towards patterns that are considered more nutritionally optimal. However, the current New Zealand dietary pattern is a poor point-of-departure given the relatively high sodium intake. Other problematic aspects of the New Zealand diet are apparent from the most recent national nutrition survey [16] and include the excessively high saturated fat intake, and being too low in: dietary fiber, potassium and some other micronutrients (e.g., selenium intake in women). Given these problems, we modeled diets by obtaining data on a wide range of individual food items and started optimizing towards a diet meeting low sodium and other nutritional requirements from there.

While avoiding the extant problems with the current New Zealand diet, a potential drawback to this bottom-up approach was the fairly modest extent to which we were able to evaluate dietary patterns that were more closely aligned to the current New Zealand diet (as has been done with optimization work around the French diet [15]). Nevertheless, we did include a range of diets with “more familiar meals”, and also included Asian, Pacific and Mediterranean-style meals.

The results obtained are also likely to have some applicability to other countries with similar types of food available and with similar dietary patterns (e.g., North America, the UK and Australia). Furthermore, because our modeling was built up from nutrient requirements rather than based on shifts from the existing New Zealand diet, this modeling can be easily adapted to address other dietary patterns by simply changing the constraints.

The model benefited from using average values from monthly pricing for many of the foods at the national level, which smoothes out seasonal variation in prices, though the low-cost fruit and vegetables selected tended to be less vulnerable to seasonal swings e.g., cabbage compared to fresh tomatoes. Another possible limitation is that we may have over-estimated the “real world” food prices given that some low-income shoppers may focus on buying “specials”, bulk buying, and only buying certain fruit and vegetables that are “local and in season”, and therefore cheaper. For example, a 10 kg bag of potatoes reduces the price by around a third of the FPI price per kg that we used. In addition, for foods not covered by the FPI, we used prices from relatively typical supermarkets and not lower-cost alternatives (e.g., from farmers’ markets or a supermarket chain that specializes in low prices). Adding to this over-estimate might be our adjustments using UK food wastage data (since New Zealanders may waste less food since they have lower average income levels relative to UK residents).

In contrast to the above, some of the estimated daily nutrient results may be on the optimistic side. Excessive home storage times and cooking may lead to loss of micronutrients. In addition, our analysis ignores complex synergies between nutrients in different foods, which are known to affect bioavailability (e.g., complementary proteins, how vitamin C enhances iron uptake, how phytates can reduce bioavailability of some minerals, etc). Certain population groups (e.g., men regularly working in hot environments) will need both higher dietary energy intakes and higher minimum sodium intakes than what we have considered in this modeling.

Nevertheless, the robustness of the study was strengthened by performing uncertainty analyses, investigating uncertainty relating to price, nutrient concentrations in foods, food wastage and nutrient requirements. The uncertainty analysis showed that even the upper SI limit for the highest sodium diet (1679 mg/d) at $9/d was still well below recommended upper limits (2300 mg/d) although not always quite meeting target upper limit levels of <1600 mg/d.

### Possible Research and Policy Implications

These results provide some reassurance that achieving such low-sodium diets is likely to be feasible when considering such factors as cost, the need to meet other nutritional recommendations, and having familiar meals to enhance acceptability. As such they can inform food selection for citizens wishing to both save food costs and to obtain relatively healthy diets. But these diets will not necessarily be favored by others who wish to spend more on food for reasons of taste or for even more optimal nutrition (e.g., consuming more fresh fruit). Some may also wish to minimize greenhouse gas emissions relating to food production by consuming even less meat and dairy products [39], [40], [41].

From a public policy perspective, a shift towards such optimized low-sodium diets and foods is likely to provide both health benefits and potentially will save health sector costs. Nevertheless, the next stage is probably for researchers to undertake health economic modeling to allow for intervention comparison. That is, to determine the most cost-effective approach or mix of approaches out of: (i) running mass media campaigns promoting these types of low-sodium diets and foods; (ii) working with celebrity chefs to make low-sodium cooking more acceptable; (iii) down-regulating permitted salt levels in processed foods (to promote food reformulation by industry); (iv) legislating for nutrition labeling on processed foods; or (v) making alternative foods more expensive by using a salt tax or other unhealthy food taxes (as per those being adopted by some countries [42]–[43])? Another policy option includes greater use of such healthy low-sodium diets and foods by institutions providing school lunches and meals in institutional settings (e.g., hospitals, retirement homes, and prisons).

## Supporting Information

File S1 **Supporting information tables.** Table S1 in File S1. Table S2 in File S1. Table S3 in File S1. Table S4 in File S1. Table S5 in File S1. Table S6 in File S1.
(PDF)
