# Supplementary material for: The Feasibility of Achieving Low-Sodium Intake in Diets That Are Also Nutritious, Low-Cost, and Have Familiar Meal Components
Source: PLoS One. 2013 Mar 7;8(3):e58539. doi: 10.1371/journal.pone.0058539 (PMC3591307; doi:10.1371/journal.pone.0058539)
Supplement: File S1 — Supporting information tables. Table S1 in File S1. Table S2 in File S1. Table S3 in File S1. Table S4 in File S1. Table S5 in File S1. Table S6 in File S1. (PDF) [file pone.0058539.s001.pdf]

Supporting Information: Additional Methods and Results

Table S1: Daily nutrient levels used for targets or constraints, with comparisons to the current New Zealand diet from the New Zealand Adult Nutrition Survey [1].[a]

Nutrient	Target or constraint values used in the modeling [2]					Comment on constraints etc	New Zealand self-reported intakes [1] (median)	
	Measure	Value for men	SD	SD as % of EAR	Value for women		Men	Women
Energy (kJ)	Minimum EER[b] level	11,450 (2734 kcal)	180	1.6%	9060	Intake must reach the EER in the modeled scenarios. Of note is that these levels may still be higher than ideal for many male New Zealanders given the high prevalence of overweight and obesity. Furthermore, the self-reported NZANS energy intakes are likely to be under-estimates [3].	10,380	7448
Saturated fatty acids (g)	Upper limit NHMRC recommenda tions	10% of energy (30 g)	2.75 g[c]	10%	20 g	Intake must be equal to or below this upper limit of 10% of daily dietary energy (as per NHMRC recommendations). Current intakes for NZ adults are considered too high.	36.5 g (total fat = 95 g)	25.8 g (total fat = 67 g)
Polyunsaturat ed fatty acids (g)	Minimum based on current NZ intake [1]	13.1 g	1.31 g[c]	10%	10 g	These current levels must be reached or exceeded. The intake of 13.1 g is equivalent to 4.8% of daily dietary energy.	13.1 g	9.6 g
Protein (g)	Minimum EAR level	52	6.24 g	12%	37	EARs must be reached/ exceeded. RDI is 64 g/d for men and 46 g/d for women.	102	71
Dietary fiber (g)	Minimum AI level	30	3 g[c]	10%	25	AI must be reached/ exceeded. No EAR has been set. Current intakes for NZ adults are considered too low.	22.1	17.5
Selected minerals								
Sodium (mg)	Upper limit	2300	230[c]	10%	2300	Upper limit must be to equal or below this level. The NHMRC Report suggests a target of 1600 mg/d (70 mmol) for men and women. Note that current intakes for NZ adults are relatively high, e.g., 2901 mg/d excluding discretionary salt [4], and 4013 mg/d based on spot urine data [5]. The lower limit was from the bottom of the NHMRC range for AI given that other authorities generally use lower values (e.g., in a WHO document the reported physiological requirement for	Not reporte d	Not reporte d
	Lower limit	460	46[c]	10%	460			

Nutrient	Target or constraint values used in the modeling [2]					Comment on constraints etc	New Zealand self-reported intakes [1] (median)	
	Measure	Value for men	SD	SD as % of EAR	Value for women		Men	Women
						sodium is 184–230 mg/d [6]).		
Potassium (mg)	Minimum AI level	3800	380[c]	10%	2800	AI must be reached/ exceeded. Current intakes for NZ adults are considered too low. The NHMRC has set no EAR or upper limit.	3449	2757
Calcium (mg)	Minimum EAR level	840	84	10%	840	EARs must be reached/ exceeded. RDI is 1000 mg/d.	919	745
Iron (mg)	Minimum EAR level	6	1.08	18%	8	In this case for the modeling we used the EAR value for women (8 mg) rather than the value for men (6 mg). RDI is 8 mg/d for men and 18 mg/d for women.	13.2	9.9
Zinc (mg)	Minimum EAR level	12	1.2	10%	6.5	EARs must be reached/ exceeded. RDI is 14 mg/d for men and 8 mg/d for women.	12.9	9.0
Selenium (µg)	Minimum EAR level	60	6	10%	50	EARs must be reached/ exceeded. RDI is 70 µg/d for men, 60 µg/d for women; current intakes for NZ women are considered too low.	67.0	47.1
Selected vitamins								
Vitamin A (µg RE)	Minimum EAR level	625	125	20%	500	EARs must be reached/ exceeded. Upper limits are 3000 µg/d for both men and women. RDI is 900 µg/d for men and 700 µg/d for women.	846	727
Vitamin B1 thiamine (mg)	Minimum EAR level	1.0	0.1	10%	0.9	EARs must be reached/ exceeded. RDI is 1.2 mg/d for men and 1.1 mg/d for women	1.6	1.1
Vitamin C (mg)	Minimum EAR level	30	6.3	21%	30	EARs must be reached/ exceeded. RDI is 45 mg/d	99	99
Vitamin E (alpha-tocopherol equivalents) (mg)	Minimum AI level	10	1[c]	10%	7	AI must be reached. No EAR has been set.	11.5	9.1

[a] Estimated Average Requirements (EARs) of nutrients/d for adults are based on values for Australia and New Zealand from the National Health and Medical Research Council (NHMRC) [2], unless otherwise stated. The NHMRC defines Estimated Average Requirement (EAR) as “a daily nutrient level estimated to meet the requirements of half of the healthy individuals in a particular life stage and gender group.” Adequate Intake (AI) is defined as “the average daily nutrient intake level based on observed or experimentally-determined approximations or estimates of nutrient intake by a group (or groups) of apparently healthy people that are assumed to be adequate.” Recommended daily intake (RDI) values are also shown where relevant. The NHMRC work did not

set an EAR for carbohydrate due to limited data. Data were based on the range for the largest adult groupings; different values may apply to adolescents and older people e.g., those aged 71+ years [2].
[b] Based on the estimated energy requirement (EER) averaged for four adult age-groups at the mid-range level of physical activity of 1.7 MJ/d [2] (1 Joule = 239 x 10^{-4} kcal).
[c] No SD available, and hence one SD was set at 10% of the EAR.
AI = adequate intake; EAR = estimated average requirements; RE = retinol equivalents; SD = standard deviation; RDI = Recommended dietary intake [2].

Table S2: Specific scenarios used for optimization modeling of low-sodium daily diets for New Zealand men.

Aim of specific scenario	Additional details on the constraints[a]
BASIC1 – Lowest sodium intake achievable	Lowest sodium intake achievable (constrained minimum 460 mg/day) while meeting all other daily energy and nutrient levels for men (Table S1). No more than 200 g of any particular vegetable or fruit (limit excluding starchy root crops: potatoes and kumara). Other daily maximum limits were: any single high-carbohydrate food (e.g., flour, pasta, rice, oats, couscous) at 120 g (1 cup of flour); homogenized milk (1 cup, 244 g); sunflower seed kernels (0.5 cup, 70 g); wheat germ (¼ cup, 29 g); coconut cream (1/4 cup, 74 g); yogurt (1/2 a commonly sold container, 250 g); and soft drinks (1 cup, 250 g). Total energy intake was set at 11,450 kJ/d.
BASIC2 – As per Scenario BASIC1 but including porridge and roti	Included a standard porridge dish at breakfast of ¼ cup (≥ 39 g) of whole grain oats and ¾ cup of milk made from ≥25 g skim milk powder.<br>Included roti (or naan) based on flour and vegetable oil at a 7:1 ratio
MED – As per Scenario BASIC1 with Mediterranean style dietary features	As per Scenario BASIC1 but with components based on data for Greek diets (median values of the EPIC study [7]) as follows:<br>• Vegetables: ≥549 g (limit excluding starchy root crops: potatoes and kumara)<br>• Fruit & nuts: ≥363 g<br>• Fish and seafood: ≥24 g<br>• Olive oil: ≥56 g i.e., ≥4 tablespoons.[b]
ASIAN – As per Scenario BASIC1 with Asian style dietary features	As per Scenario BASIC1 but with these additional components:<br>(i) rice (≥200 g);<br>(ii) vegetable oil for stir-fry cooking (1 tablespoon = 14 g);<br>(iii) vegetables ≥500 g with minimum amounts of: carrots (≥50 g); cabbage (≥50 g); broccoli (≥50 g); onion (≥50 g); and Chinese cabbage ["bok choy"] (≥50 g). The 200 g maximum for any particular vegetable also applied.
NZ-Meat1 – As per Scenario BASIC1 with New Zealand style Budget meat meal 1 (mince)	As per Scenario BASIC1 but with the following components:<br>• Evening meal: "Mum's mince on toast" recipe (NZ Beef & Lamb recipe website):<br>o beef mince [≥125 g]; onion [≥28 g], carrot [≥15 g], any other vegetable [≥40 g], wholemeal flour [≥8 g], 1 slice of bread [≥28 g]<br>o condiments/sauces excluded.<br>• Standard breakfast: Porridge<br>o As per Scenario BASIC2 but with 0.5 tablespoon sugar (≥7 g).<br>• Standard lunch:<br>o Half a cheese sandwich (with a whole sandwich comprising: 2 slices of wholemeal bread [28 g slices x 2]; ≥24 g of mild cheddar cheese; ≥10 g of margarine)<br>o Half a peanut butter sandwich (with a whole sandwich comprising: 2 slices of wholemeal bread [28 g slices x 2]; ≥25 g of peanut butter;

Aim of specific scenario	Additional details on the constraints[a]
	≥10 g of margarine)<br>○ 1 apple (≥130 g).
NZ-Meat2 – As per Scenario BASIC1 with New Zealand style Budget meat meal 2 (sausages)	As per Scenario BASIC1 but with but the following components:<br>• Sausages (≥96 g, 2 servings); ≥426 g of peeled potatoes (2 medium potatoes); and any two other vegetables of at least 100 g per vegetable (limit excluding the starchy root crops of: potatoes and kumara).<br>• Dessert: ice cream (≥66 g [0.5 cups]); canned peaches, fruit salad or apricots (with syrup/juice) ≥147 g.<br>• Standard breakfast and lunch as per NZ-Meat1
NZ-Fish – As per Scenario BASIC1 with New Zealand style Budget fish meal (tuna pasta bake)	As per Scenario BASIC1 but with the following components:<br>• Canned tuna in spring water: ≥124 g (drained weight), pasta [≥118 g, dry weight]; ≥120 g [0.5 cup] of canned tomatoes; and at least 100 g of any other prepared vegetable (limit excluding starchy root crops: potatoes and kumara).<br>• Standard breakfast and lunch as per NZ-Meat1
NZ-Pacific – As per Scenario BASIC1 with "Pacific style" evening meal	As per Scenario BASIC1 but with the following components:<br>• Taro root (peeled) ≥104 g (1 cup of cubes); canned tuna in spring water ≥77 g (0.5 cup drained weight); "lite" coconut cream ≥222 g [0.75 cups] (or for women: 0.5 cup, 148 g); onion (peeled) ≥14 g; vegetable oil ≥7 g (0.5 tablespoon)[c]<br>• Standard breakfast and lunch as per NZ-Meat1

[a] All the weights in this table are for prepared ready-to-eat items, with purchased weights assumed to be higher due to inedible portions such as skins and spoiled parts (wastage proportions were obtained from the USDA database) [8].

[b] For simplicity we did not define other components of the Mediterranean diet relating to: legumes, cereals, dairy products, meat products, and alcohol (ethanol).

[c] Proportions based on the recipe for a "taro and seafood" dish (http://www.fao.org/WAIRdocs/x5425e/x5425e01.htm). Taro leaves were not included as these may be less readily available in New Zealand.

Table S3: Food products included in the optimization modeling and related input data (price and wastage factors).[a]

Foods	Details	Price source	Price (NZ$) per 100 g	SD on price (NZ$)	Total wastage (%)[b]	SD on wastage (%)
Fruit and vegetables						
Potatoes		FPI 2011	0.17	0.04	45	1.42
Cabbage		FPI 2011	0.19	0.08	46	1.98
Carrots		FPI 2011	0.22	0.03	46	1.86
Canned tomatoes		Countdown online	0.24	0.01	14	1.18
Chinese cabbage	Including bok choy, pak choy	Countdown online (seasonally adjusted)	0.25	0.06	46	1.98
Canned fruit salad	Juice included	Countdown online	0.26	0.01	16	1.26
Apples		FPI 2011	0.26	0.08	46	2.09
Canned apricot halves	Juice included	Countdown online	0.27	0.01	16	1.26
Frozen peas		FPI 2011	0.29	0.01	14	1.37
Kiwifruit, green		FPI 2011	0.34	0.29	40	1.84
Kumara		Countdown online	0.38	0.08	46	2.09
Bananas	Edible portion (64% of weight; USDA)	FPI 2011	0.42	0.07	43	1.39
Canned peaches	Juice included	FPI 2011	0.42	0.03	16	1.23
Oranges	Edible portion (68% of weight; USDA)	FPI 2011	0.46	0.03	40	1.67
Onions		Countdown online	0.46	0.10	46	1.73
Taro (white)		Countdown online	0.50	0.11	46	2.14
Lettuce		FPI 2011	0.52	0.51	55	2.03
Sultanas		FPI 2011	0.57	0.02	16	1.26
Broccoli		FPI 2011	0.59	0.29	46	2.58
Tomatoes		FPI 2011	0.65	0.56	46	2.09
Raisins		Countdown online	0.79	0.03	16	1.26
Mushrooms		FPI 2011	1.07	0.08	46	2.35
Cereals and grains						
White flour		FPI 2011	0.14	0.01	16	1.33

Foods	Details	Price source	Price (NZ$) per 100 g	SD on price (NZ$)	Total wastage (%)[b]	SD on wastage (%)
Wholemeal flour		Countdown store	0.14	0.01	16	1.33
Pasta	Generic brand dry spaghetti	Countdown online	0.21	0.01	16	1.47
Rice (white)	Long grain white	FPI 2011	0.25	0.01	16	1.33
White bread		FPI 2011	0.29	0.01	36	1.27
Oats (wholegrain)	Generic brand quick oats, 100% wholegrain	Countdown store	0.32	0.01	16	1.50
Semolina		Countdown online	0.38	0.02	25	1.58
Wholemeal bread	Branded wholemeal bread	Countdown online	0.40	0.02	36	1.70
Canned spaghetti		FPI 2011	0.40	0.02	16	1.26
Breakfast wheat biscuits	"Weetbix" or similar products	FPI 2011	0.55	0.02	16	1.44
Pop corn		New World bulk bin	0.79	0.04	25	1.84
Wheat germ		Countdown online	0.92	0.04	25	1.84
Couscous		New World bulk bin	0.99	0.05	25	1.84
Pulses, seeds and nuts						
Dry peas	Split peas (green)	Countdown online	0.34	0.01	14	1.37
Chickpeas	Canned	Countdown online	0.60	0.02	14	1.37
Canned lentils	Canned low-salt (drained weight)	Countdown online	0.65	0.03	14	1.18
Lentils (dried)	Dried brown or split red lentils	New World bulk bin	0.99	0.04	14	1.37
Peanuts	Raw, blanched	New World bulk bin	1.09	0.05	16	1.47
Sunflower seeds	Hulled seeds	New World bulk bin	1.29	0.05	16	1.47
Fish and meat						
Luncheon sausage	Pork flavor, sliced	Countdown online	0.59	0.03	23	1.40
Sausages	Mix of meats	FPI 2011	0.86	0.04	23	1.40
Beef mince		FPI 2011	1.19	0.05	23	1.63
Sardines	In spring water (drained weight)	Countdown online	1.22	0.06	23	1.55
Tuna (canned)	Price based on	FPI 2011	1.30	0.09	23	1.55

Foods	Details	Price source	Price (NZ$) per 100 g	SD on price (NZ$)	Total wastage (%)[b]	SD on wastage (%)
	drained weight for generic brand tuna in spring water					
Beef, blade steak		FPI 2011	1.42	0.06	23	1.63
Lamb, neck chops		FPI 2011	1.55	0.11	23	1.63
Pork mince		Countdown online	1.60	0.07	23	1.40
Canned ham		Countdown online	1.66	0.08	23	1.52
Canned sheep meat		Countdown online	1.67	0.08	23	1.52
Pork, loin chops		FPI 2011	1.68	0.05	23	1.63
Pie, steak		FPI 2011	1.96	0.03	35	1.72
Canned chicken		Countdown online	2.11	0.12	38	1.95
Pork, bacon		FPI 2011	2.16	0.06	23	1.40
Beef, sirloin steak		FPI 2011	2.52	0.13	23	1.63
Dairy products						
Milk (whole, homogenized)		FPI 2011	0.18	0.00	7	0.49
Ice cream	Vanilla, low fat	Countdown online	0.30	0.01	9	0.95
Yogurt		FPI 2011	0.60	0.02	9	0.90
Butter	Salted	FPI 2011	0.87	0.05	9	0.95
Milk powder	Generic brand skim milk powder	Countdown online	1.00	0.04	7	0.77
Cheese	Mild cheddar	FPI 2011	1.02	0.06	9	0.87
Soft drink		FPI 2011	0.16	0.01	9	1.06
Sugar	White sugar	FPI 2011	0.20	0.01	16	1.30
Salt	Table salt	Countdown online	0.24	0.01	35	1.92
Juice	Crisp Apple juice	FPI 2011	0.31	0.14	14	1.27
Coconut cream ("lite")		Countdown online	0.36	0.02	25	1.58
Vegetable oil	Canola or blended vegetable oil	Countdown online	0.47	0.02	17	1.30
Tomato sauce		FPI 2011	0.52	0.03	14	1.21
Eggs	Medium sized eggs	FPI 2011	0.54	0.01	9	0.76

Foods	Details	Price source	Price (NZ$) per 100 g	SD on price (NZ$)	Total wastage (%)[b]	SD on wastage (%)
Peanut butter	Generic brand peanut butter smooth	Countdown online	0.61	0.03	16	1.26
Margarine	Reduced salt	Countdown online	0.74	0.03	17	1.30
Olive oil		Countdown online	1.10	0.05	17	1.30
Potato crisps		FPI 2011	1.17	0.19	6	0.73
Biscuit	Chocolate coated	FPI 2011	1.55	0.06	6	0.88
Chocolate	Dark	FPI 2011	1.55	0.04	6	0.83

SD = Standard deviation.
[a] All fruit and vegetables were fresh and raw unless otherwise stated. Prices were obtained from the Food Price Index (FPI) or from Countdown or New World supermarkets. The FPI is based on average monthly prices from supermarkets and greengrocers over a 12-month period.
[b] Given the absence of NZ food wastage data we used data from a large UK study (WRAP study) [9]. This provided wastage data on some specific foods but also for the more general food categories (e.g., “fresh fruit”) as per Tables 51 and 52 in the Report on this study. We used total wastage in the calculations – so that if there was 50% wastage of a food the effective cost per food nutrient gained was doubled. This total wastage includes both non-edible parts of the food (e.g., banana skins) but also potentially edible food that is not eaten (e.g., if it spoils before preparation or is otherwise discarded). In some cases, the values for total waste had to use the very general category of “staple” (16% waste) or the national average for all food in the UK (25% waste).

Table S4: Foods selected by the optimization process for men for low-sodium for the various daily dietary scenarios with a daily cost constraint of <NZ$15/d.

Food category[a] and foods	Total food weights suggested per day (g) by scenario							
	BASIC1	BASIC2	MED	ASIAN	NZ-Meat1	NZ-Meat2	NZ-Fish	NZ-Pacific
**Vegetables**								
Mushrooms	200	200	88	140	0	0	0	0
Tomatoes (fresh)	25	0	0	0	200	0	200	200
Carrots	21	29	25	200	15	200	10	10
Chinese cabbage	0	0	200	150	0	0	0	0
Lettuce	0	0	200	0	0	0	0	0
Cabbage	0	0	36	50	0	0	0	0
Broccoli	0	0	0	50	0	0	0	0
Onions	0	0	0	50	28	0	0	14
Frozen peas (F)	0	0	0	0	38	0	0	106
Tomatoes (C)	0	0	0	0	0	0	120	0
**Total**	**247**	**229**	**549**	**640**	**281**	**200**	**330**	**330**
**Starchy vegetables**								
Potatoes	158	132	0	0	0	426	0	0
Taro	0	0	0	0	0	0	0	500
**Fruit**								
Oranges	0	20	200	0	200	0	133	0
Apples	0	0	0	0	130	130	130	130
Kiwifruit (green)	0	0	163	0	200	0	200	0
Apricots (C)	0	0	0	0	200	147	200	200
Peaches (C)	0	0	0	0	0	0	15	73
**Total**	**0**	**20**	**363**	**0**	**730**	**277**	**678**	**403**
**Cereals and grains[b]**								
Couscous	120	120	120	120	120	120	120	84
Semolina	120	73	120	62	0	0	0	0
Pasta	120	0	120	85	0	0	118	0
Oats	90	107	120	18	66	39	39	39
Rice (white)	73	120	29	240	0	0	0	0
Flour (wholemeal)	10	0	0	0	86	0	103	75
Flour (white)	0	120	0	0	0	112	0	0
Pop corn	0	0	23	0	0	0	0	0
Bread	0	0	0	0	84	56	56	56
Wheat germ	0	0	0	0	29	0	29	0
**Total**	**533**	**540**	**532**	**526**	**386**	**327**	**465**	**254**
**Pulses, seeds and nuts**								

Food category[a] and foods	Total food weights suggested per day (g) by scenario							
	BASIC1	BASIC2	MED	ASIAN	NZ-Meat1	NZ-Meat2	NZ-Fish	NZ-Pacific
Sunflower seeds	15	15	8	13	70	70	70	70
**Meat/fish**								
Beef steak	9	0	0	0	0	0	0	0
Chicken (C)	0	78	0	0	0	0	0	0
Tuna (C)	0	0	24	0	0	0	124	77
Beef mince	0	0	0	94	125	0	0	0
Sausages	0	0	0	0	0	96	0	0
**Total**	**9**	**78**	**24**	**94**	**125**	**96**	**124**	**77**
**Dairy**								
Milk (homogenized, whole)	244	244	151	244	0	0	0	0
Ice cream	48	4	0	0	0	66	0	0
Milk powder (skim)	21	25	0	15	25	25	25	25
Cheese	0	0	34	13	12	12	12	12
**Total**	**313**	**273**	**185**	**272**	**37**	**103**	**37**	**37**
**Added fat, spreads**								
Olive oil	30	17	56	0	0	0	0	0
Butter	31	23	0	0	0	0	0	0
Coconut cream	0	47	0	74	0	0	0	222
Vegetable oil	0	0	0	14	10	25	0	7
Peanut butter	0	0	0	0	13	13	13	13
Margarine	0	0	0	0	10	10	10	10
**Total**	**61**	**87**	**56**	**88**	**33**	**47**	**23**	**252**
**Other**								
Chocolate, dark	0	0	0	28	0	0	0	0
Sugar (added)	0	0	0	0	7	7	7	7
**Total food weights per day (g)**	**1,336**	**1,373**	**1,718**	**1,660**	**1,668**	**1,553**	**1,734**	**1,929**
**Number of food items**	**17**	**17**	**18**	**19**	**21**	**17**	**21**	**21**

[a] Presented in descending order of quantity (starting with Scenario BASIC1).
[b] Wholemeal or wholegrain unless otherwise stated.
C = canned; F = frozen

Table S5: Key nutrient results for the optimization process for men when minimizing sodium intake but with a more relaxed daily food cost constraint (<NZ$15/d).

**Scenario**[a]	**Cost (NZ$/d)**	**Energy (kJ)**	**Potassium (mg)**	**Sodium**[b] **(mg)**	**Potassium / Sodium (ratio)**
BASIC1	10.02	11,450	3,800	460	8.3
BASIC2	11.84	11,450	3,800	460	8.3
MED	12.41	11,450	3,838	460	8.3
ASIAN	11.45	11,450	3,800	460	8.3
NZ-Meat1	12.82	11,450	4,945	899	5.5
NZ-Meat2	8.66	11,450	5,024	1,641	3.1
NZ-Fish	12.64	11,450	4,969	1,096	4.5
NZ-Pacific	14.99	11,450	5,781	886	6.5
Constraints (nutrients for men)	**<15.00**	**≥11,450**	**≥3800**	**≥460 and ≤2300**	**Nil**

[a] See Table S2 for explanation of the dietary scenarios.

[b] Values represent the objective function value in each scenario (i.e., the key value being minimized in the optimization process).

[c] This scenario required a slightly higher cost constraint to allow for an optimal solution.

Table S6: Uncertainty and heterogeneity analysis for sodium and other nutrient intakes as a result of the optimization process for men for two of the daily dietary scenarios.

Nutrients (point estimate for constraint – but without uncertainty)	A low sodium intake scenario (BASIC1) selected foods (g/day) (2000 iterations)				The highest sodium intake scenario (NZ-Meat2) selected foods (g/day) (403 iterations)			
	**Mean**	**Median**	**Lower 95%SI bound**	**Upper 95%SI bound**	**Mean**	**Median**	**Lower 95%SI bound**	**Upper 95%SI bound**
Energy (=11,450 kJ)	11,458	11,454	11,123	11,803	11,444	11,445	11,110	11,773
Saturated fatty acids (≤30 g)[b]	28	28	19	34	21	22	17	26
Polyunsaturated fatty acids [PFA] (≥13.1 g)	43	43	32	58	44	46	30	58
Protein (≥52 g)	93	92	69	124	91	88	76	109
Total sugars (g)	123	121	79	185	103	100	90	128
Dietary fiber (≥30 g)[b]	32	32	26	38	40	37	31	60
**Minerals (selected)**								
Sodium (≤2300 mg)	460	460	460	460	1,644	1,640	1,551	1,735
Potassium (≥3800 mg)	3,883	3,873	3,276	4,553	5,345	5,248	4,730	6,334
Calcium (≥840 mg)[b]	1,036	1,045	704	1,350	851	836	773	997
Iron (≥8 mg)	14	14	11	16	18	17	14	26
Zinc (≥12 mg)[b]	13	13	11	16	15	13	11	21
Selenium (≥60µg)[b]	68	68	60	78	65	67	49	75
**Vitamins (selected)**								
Vitamin A (≥625 & ≤3,000 µg RE)[b]	627	629	377	862	2,378	2,380	2,169	2,578
Thiamine (≥1 mg)	2.8	2.7	2.2	3.9	3.4	3.3	2.5	4.3
Vitamin C (≥30 mg)[b]	49	47	23	88	122	105	86	276
Vitamin D (mcg)	1	1	0	3	2	2	2	2
Vitamin E (≥10 mg)	40	38	33	52	38	38	32	44
**Calculated ratios**								
PFA/ saturated fats ratio[c]	1.5	1.5	1.6	1.7	2.0	2.1	1.7	2.3
Potassium/sodium ratio[c]	8.4	8.4	7.1	9.9	3.3	3.2	3.1	3.7
**Cost** (NZ$) per day	8.36	8.43	7.28	8.99	9.55	9.56	8.81	10.00

SI = simulation intervals from probabilistic distributions for both uncertainty and heterogeneity.

[a] For this scenario the daily food cost limit had to be raised (up to $10/d) to increase the number of feasible solutions. Even so, the number of feasible solutions was constrained given that we decided to maintain all the upper limits for foods used in the deterministic modeling and the full range of uncertainty and heterogeneity possible (i.e., for nutrient concentrations, nutrient requirements, food prices and food wastage levels).

[b] Some of the results in these rows do not reach the stated nutrient constraints because the nutrient requirements were also subject to uncertainty as part of the uncertainty analysis (see *Methods*).

[c] Ratios of mean (and median and SI), not the mean ratio.

Table S7: Foods selected by the optimization process for low-sodium for the various daily dietary scenarios with a daily cost constraint of <NZ$9/d and for nutritional requirements for adult women.

Food category[a] and foods	Total food weights suggested per day (g) by scenario							
	BASIC1	BASIC2	MED	ASIAN	NZ-Meat1	NZ-Meat2	NZ-Fish	NZ-Pacific
**Vegetables**								
Mushrooms	60	113	0	0	0	0	0	0
Carrots	23	21	14	150	17	200	10	23
Broccoli	13	21	0	50	134	0	0	0
Cabbage	6	0	0	50	200	0	0	155
Chinese cabbage	0	0	200	50	0	0	0	0
Lettuce	0	0	200	0	0	0	0	0
Peas (F)	0	0	135	0	0	0	172	0
Onions	0	0	0	200	28	0	0	14
Tomatoes (C)	0	0	0	0	0	0	120	0
Tomatoes (fresh)	0	0	0	0	0	0	56	0
**Total**	**103**	**154**	**549**	**500**	**378**	**200**	**358**	**192**
**Starchy vegetables**								
Potatoes	113	28	0	0	0	426	0	0
Taro	0	0	0	0	0	0	0	223
**Fruit**								
Oranges	0	0	200	0	72	0	200	200
Kiwifruit (green)	0	0	163	0	0	111	137	0
Apples	0	0	0	0	130	130	130	130
Apricots (C)	0	0	0	0	0	147	0	200
**Total**	**0**	**0**	**363**	**0**	**202**	**388**	**467**	**530**
**Cereals and grains[b]**								
Oats	120	120	120	78	120	39	120	120
Couscous	120	120	0	120	104	108	0	3
Flour (white)	120	109	54	0	0	0	0	0
Pasta	113	120	120	0	0	0	118	0
Semolina	0	18	0	0	0	0	0	0
Rice (white)	0	0	0	200	0	0	0	0
Flour (wholemeal)	0	0	0	0	120	0	28	120
Bread	0	0	0	0	84	56	56	56
**Total**	**473**	**488**	**294**	**398**	**344**	**147**	**266**	**243**
**Pulses, seeds and nuts**								
Sunflower seeds	9	9	40	0	20	70	70	11

Food category[a] and foods	Total food weights suggested per day (g) by scenario							
	BASIC1	BASIC2	MED	ASIAN	NZ-Meat1	NZ-Meat2	NZ-Fish	NZ-Pacific
**Meat/fish**								
Tuna	0	0	0	0	0	0	124	77
Sardines	0	0	28	0	0	0	0	0
Sausages	0	0	0	0	0	96	0	0
Beef mince	0	0	0	0	125	0	0	0
**Total**	**0**	**0**	**28**	**0**	**125**	**96**	**124**	**77**
**Dairy**								
Milk powder (skim)	0	49	3	0	25	25	25	25
Cheese	28	0	23	13	12	12	12	12
Ice cream	0	0	0	0	0	66	0	0
Milk (homogenized, whole)	34	0	0	138	0	0	0	0
Yogurt	250	0	50	207	18	0	0	0
**Total**	**312**	**49**	**76**	**358**	**55**	**103**	**37**	**37**
**Added fat, spreads**								
Olive oil	22	13	56	25	0	0	0	0
Butter	13	28	0	0	0	0	0	0
Vegetable oil	0	2	0	14	0	0	0	7
Coconut cream	0	0	0	62	0	0	0	148
Peanut butter	0	0	0	0	13	13	13	13
Margarine	0	0	0	0	10	10	10	10
**Total**	**35**	**44**	**56**	**101**	**23**	**23**	**23**	**178**
**Other**								
Eggs	0	0	0	83	0	0	0	0
Sugar (added)	0	0	0	0	7	7	7	7
**Total food weights consumed per day (g)**	**1,045**	**772**	**1,406**	**1,453**	**1,239**	**1,516**	**1,408**	**1,555**
**Number of food items**	**15**	**14**	**15**	**16**	**18**	**16**	**18**	**20**

[a] Presented in descending order of quantity (starting with Scenario BASIC1).
[b] Wholemeal or wholegrain unless otherwise stated.
C = canned; F = frozen

Table S8: Sodium and other nutrient intakes for the optimal solution for the various daily dietary scenarios with a cost constraint of <NZ$9/d and for the nutrient recommendations for adult women (where sodium is the objective function value in each scenario).

	**Dietary scenario**							
**Nutrients (constraints)**	**BASIC1**	**BASIC2**	**MED**	**ASIAN**	**NZ-Meat1**	**NZ-Meat2**	**NZ-Fish**	**NZ-Pacific**
Energy (=9060 kJ)[a]	9,060	9,060	9,060	9,060	9,060	9,060	9,060	9,060
Saturated fatty acids (≤20 g)[a]	20	20	20	20	20	17	12	20
Polyunsaturated fatty acids (≥10 g)[a]	10	10	24	12	16	30	31	14
Protein (≥37 g)[a]	77	81	75	71	102	82	116	80
Total sugars (g)	14	25	43	41	60	105	72	120
Dietary fiber (≥25 g)[a]	25	25	42	25	52	37	51	58
**Selected minerals**								
Sodium (≥460 & ≤2300 mg)[a]	460	460	460	460	918	1,642	1,101	896
Potassium (≥2800 mg)[a]	2,800	2,800	3,408	2,800	3,704	5,151	3,872	4,302
Calcium (≥840 mg)	840	840	840	840	840	840	840	840
Iron (≥8 mg)	15	15	19	11	23	16	23	20
Zinc (≥6.5 mg)[a]	11	11	11	9	19	12	15	20
Selenium (≥50 µg)[a]	50	50	50	50	50	64	115	56
**Selected vitamins**								
Vitamin A (≥500* & ≤3,000 µg RE)	500	500	500	1,906	500	2,300	500	500
Thiamine (≥0.9 mg)[a]	2	2	2	1	3	3	4	2
Vitamin C (≥30 mg)	30	30	293	94	215	175	255	157
Vitamin D (mcg)	1	1	3	2	2	2	7	5
Vitamin E (≥7 mg)[a]	7	7	22	7	14	33	34	19
**Calculated ratios**								
Polyunsaturated/saturated fats ratio	0.5	0.5	1.2	0.6	0.8	1.7	2.6	0.7
Potassium/sodium ratio[b]	6.1	6.1	7.4	6.1	4.0	3.1	3.5	4.8
**Cost** (<$NZ9)	6.92	6.38	8.99	8.99	8.99	8.81	8.99	8.99

[a] Nutrient recommendations lower than for men (see Table S1).

[b] Ratios of mean (and median and SI), not the mean ratio.

References

1. University of Otago and Ministry of Health. (2011) A Focus on Nutrition: Key findings of the 2008/09 New Zealand Adult Nutrition Survey Wellington: Ministry of Health.
2. National Health and Medical Research Council, Ministry of Health. (2006) Nutrient reference values for Australia and New Zealand. Commonwealth of Australia. Available: http://www.nhmrc.gov.au/_files_nhmrc/publications/attachments/n35.pdf?q=publications/synopses/_files/n35.pdf Accessed 12 June 2012.
3. Pikholz C, Swinburn B, Metcalf P. (2004) Under-reporting of energy intake in the 1997 National Nutrition Survey. N Z Med J 117: U1079.
4. Ministry of Agriculture and Forestry. (2011) 2009 New Zealand Total Diet Study: Agricultural compound residues, selected contaminant and nutrient elements Wellington: Ministry of Agriculture and Forestry, 2011. Available: http://foodsafety.govt.nz/science-risk/programmes/total-diet-survey.htm Accessed 12 June 2012.
5. McLean R, Williams S, Mann J, Parnell W. (2011) How much salt are we eating? Estimates of New Zealand population sodium from the 2008/2009 Adult Nutrition Survey [Presentation on 2 December 2011]. Joint Annual Scientific Meeting of the Australian and New Zealand Nutrition Societies. Queenstown, New Zealand (29 November - 2 December), 2011.
6. Elliot P, Brown I. (2007) Sodium intakes around the world Geneva: World Health Organization. Available: http://www.who.int/dietphysicalactivity/Elliot-brown-2007.pdf Accessed 1 December 2012.
7. Trichopoulou A, Bamia C, Trichopoulos D. (2009) Anatomy of health effects of Mediterranean diet: Greek EPIC prospective cohort study. BMJ 338: b2337.
8. USDA. (2011) USDA National Nutrient Database for Standard Reference. Available: http://www.ars.usda.gov/Services/docs.htm?docid=22112 Accessed 12 June 2012.
9. WRAP. (2009) Household Food and Drink Waste in the UK. Banbury: Report prepared by WRAP. Available: http://www.wrap.org.uk/downloads/Household_Food_and_Drink_Waste_in_the_UK_Nov_2011.fa0a205b.8048.pdf; Accessed 12 June 2012.
